# Gap junctional protein Cx43 is involved in the communication between extracellular vesicles and mammalian cells

**DOI:** 10.1038/srep13243

**Published:** 2015-08-19

**Authors:** Ana Rosa Soares, Tania Martins-Marques, Teresa Ribeiro-Rodrigues, Joao Vasco Ferreira, Steve Catarino, Maria João Pinho, Monica Zuzarte, Sandra Isabel Anjo, Bruno Manadas, Joost P.G. Sluijter, Paulo Pereira, Henrique Girao

**Affiliations:** 1 Centre of Ophthalmology and Vision Sciences, Institute of Biomedical Imaging and Life Sciences (IBILI), Faculty of Medicine, University of Coimbra, Azinhaga de Sta Comba, 3000-354 Coimbra, Portugal; 2 CNC - Center for Neuroscience and Cell Biology, University of Coimbra, 3004-517 Coimbra, Portugal; 3 Department of Life Sciences, Faculty of Sciences and Technology, University of Coimbra, 3004-517 Coimbra, Portugal; 4 Department of Cardiology, Division of Heart & Lungs, University Medical Center Utrecht, the Netherlands; 5 Interuniversity Cardiology Institute Netherlands (ICIN), Utrecht, the Netherlands

## Abstract

Intercellular communication is vital to ensure tissue and organism homeostasis and can occur directly, between neighbour cells via gap junctions (GJ), or indirectly, at longer distances, through extracellular vesicles, including exosomes. Exosomes, as intercellular carriers of messenger molecules, mediate the transfer of biological information between donor and acceptor cells. Although the biological effects of exosomes in target cells have been intensively studied, the mechanisms that govern exosomal uptake are not fully understood. Here, we show that Connexin 43 (Cx43), the most widely expressed GJ protein, is present in exosomes in the form of hexameric channels and, more importantly, that exosomal Cx43 is able to modulate the interaction and transfer of information between exosomes and acceptor cells. This study envisions a new paradigm where Cx43-containing channels mediate the release of exosomal content into cells, which constitutes a novel and unanticipated mechanism to modulate intercellular communication.

A well-regulated and efficient communication between cells is vital to ensure homeostasis and survival of multicellular organisms. Intercellular communication can occur directly, between neighbour cells via gap junctions (GJ), or indirectly at longer distances through soluble factors and extracellular vesicles (EVs) released into the environment. According to their size, composition and subcellular origin, EVs can be divided into apoptotic bodies, microvesicles (MVs) and exosomes^1^,^2^,^3^. Although initially regarded as by-products of uncontrolled cell disposal, nowadays exosomes, that originate from the fusion of multivesicular bodies (MVB) with the plasma membrane, are considered intercellular messengers, capable of mediating local and systemic cell communication^4^,^5^,^6^,^7^. To elicit a cell response, exosomes have to dock and be taken up by the acceptor cells, in a process that relies, at least partially, on protein-protein interactions^8^,^9^ via e.g. the tetraspanins CD9, CD63 and CD81 or the Integrin alpha v beta 3 (Vitonectin receptor)^10^. However, given the complexity and specificity of this mechanism, it is likely that other proteins are involved in the docking, fusion and/or internalization of exosomes by target cells. In this work we hypothesize that exosomes can interact with target cells in a similar way as neighbouring cells communicate with each other, that is, through Connexin(Cx)-containing channels, that allow the passage of small substances (<1 kDa) such as second messengers, ions, metabolites and genetic material between adjacent cells^11^,^12^. Cx43, the most widely expressed Cx, oligomerizes into hexameric channels in the ER which are subsequently transported to the plasma membrane, where they dock with opposing hemichannels of neighbour cells to form GJ plaques, through which intercellular communication occurs. This communication can be regulated at different levels, namely channel gating, Cx43 synthesis, trafficking and degradation^13^. Studies from our group established that ubiquitination of Cx43 signals GJ internalization and degradation^14^,^15^,^16^, which results in down regulation of intercellular communication. The results obtained in this study demonstrate that Cx43 is present in exosomes as hexameric channels and more importantly, beyond cell-cell communication, Cx43 is able to modulate the interaction and communication between exosomes and cells. In conclusion, our data ascribes a novel and unanticipated biological role for Cx43 in mediating the transfer of information between exosomes and acceptor cells.

## Results

### The gap junctional protein Cx43 is present in exosomes isolated from cultured cells and biological fluids

**Figure 1:**
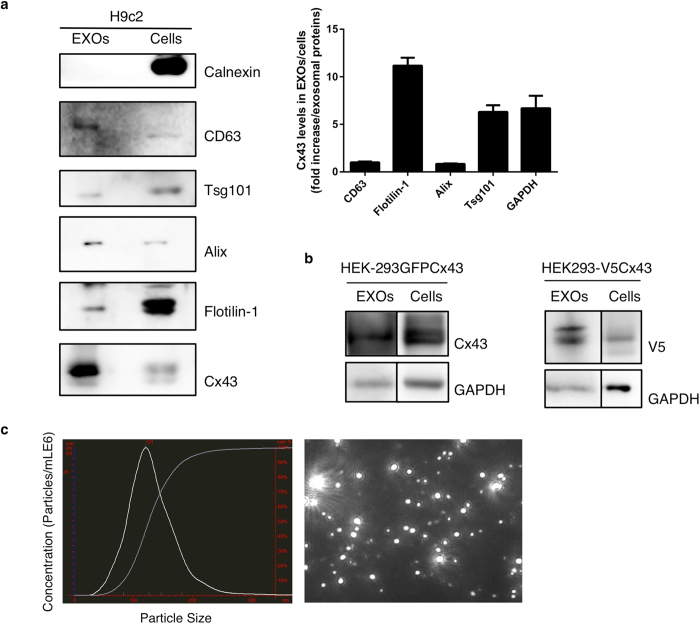
Gap junctional protein Cx43 is present in exosomes isolated from cultured cells. (**a**) Exosomes were isolated from H9c2 by differential ultracentrifugation, after which WB analysis was performed in exosomal (EXOs) and cell lysates to assess the presence of Cx43 (AB0016) and exosomal markers CD63 (AB0047), Tsg101, Alix and Flotillin-1. 15 μg exosomal and 30 μg cell extracts were used. Calnexin was used as control for cell debris contamination. Values on graph show the ratio of Cx43 levels in exosomes/cells as fold increase over the levels of the indicated exosomal protein levels. (**b**) WB analysis of Cx43 in EXOs from HEK-293GFPCx43 and HEK-293V5Cx43 cells. 5 μg exosomal and 30 μg cell extracts were used. (**c**) Nanosight tracking analysis (NTA) was performed in 5 μg exosomes isolated from H9c2 cells. Representative graph of exosomal concentration (particles/ml^6^) and size distribution (left panel) as measured by NTA. Right panel depicts a screen shot of video from NanoSight LM10 showing optimal light scatter.

Given the lack of consensus in the literature regarding the nomenclature adopted to refer to the different EVs, it should be noted that when using the term exosomes, these may represent a larger set of EVs. In this study, we hypothesized that channels formed by Cx43 mediate communication between exosomes and cells. In accordance with this hypothesis, we first investigated the presence of Cx43 in exosomes obtained from various sources. For this purpose, we isolated exosomes released by different types of cells that endogenously express Cx43, including the heart cell line H9c2 (Fig. 1a), the retinal pigment epithelial cell line ARPE-19 (data not shown), and HEK-293 stable cell lines over-expressing GFP-labelled Cx43 (GFP-Cx43) or V5-tagged Cx43 (V5-Cx43) (Fig. 1b). Exosomes were isolated from cell culture supernatants by differential ultracentrifugation after incubation for 24 h in exosome-free medium. The presence of Cx43 was further determined by Western Blot (WB). Results presented in Fig. 1a,b show that the analyzed cell lines released exosomes containing Cx43. To further confirm that the isolation procedure employed gave rise to a vesicle population highly enriched in exosomes, we used nanosight tracking analysis (NTA) to assess the size of the vesicles and WB to evaluate the presence of exosomal markers. Calnexin was used to demonstrate that the isolated exosomes were free of cytoplasmic protein contamination (Fig. 1a). For each cell line, we evaluated the relative abundance of Cx43 in exosomes, in comparison with proteins previously described as exosomal markers. The results obtained show that for all three cell lines tested, exosomes are particularly enriched in Cx43, in comparison with Flotillin-1, Tsg101 and GAPDH (Fig. 1a, Supplementary Fig. 1a,b). Additionally, our data showed that EV-enriched extracts containing Cx43 are detected with an approximate vesicle size between 80 and 200 nm (Fig. 1c). These results support the idea that EVs, including exosomes, contain Cx43.

**Figure 2:**
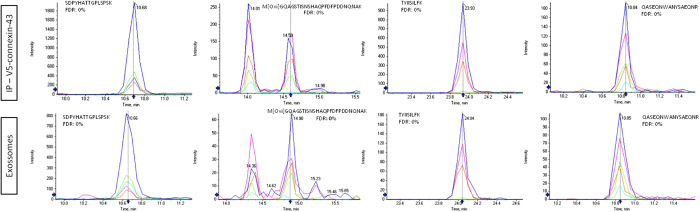
Detection of Cx43 in exosomes by targeted-SWATH-MS. Peak groups (extracted ion traces of chosen fragments) of Cx43 peptides previously identified in IDA experiments for both a positive control using a Cx43 enriched sample (top panel), and for the exosomal sample (bottom panel). For each peak group (peptide), the peptide sequence and FDR value obtained using SWATHTM processing plug-in for PeakView is presented.

Although plasma membrane proteins have previously been identified in exosomes, Cx43 has never been reported in proteomic analyses of exosomes. To address this issue, a targeted-SWATH-MS method was built for a set of Cx43-specific peptides previously identified in a Cx43-immunopurified sample^17^. Figure 2 shows that, of this set of screened peptides, 4 peptides (corresponding to 17.3% coverage of the Cx43 sequence) are detected in both a positive control (a Cx43-immunopurified sample) and in the exosome sample. Besides the presence of Cx43, our proteomic analysis also identified other exosomal proteins, such as CD63, Alix, Hsc70, Hsp90, GAPDH and actin (Supplementary Table S1), thereby confirming that our EV-extract, where Cx43 is detected, is enriched in exosomes.

**Figure 3:**
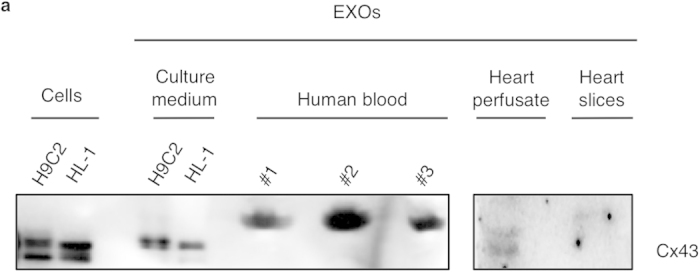
Gap junctional protein Cx43 is present in exosomes isolated from various biological fluids. (**a**) Exosomes were isolated from H9c2, HL-1 cells, organotypic heart cultures and human plasma. After 30 min of perfusion in a Langendorff apparatus, rat heart perfusates were collected and exosomes were further isolated. WB analysis was performed in exosomes (EXOs) and cell lysates. #1, 2 and 3 represent different human donors.

Besides cell culture supernatants from cardiac cell lines H9c2 and HL-1 cells, we also demonstrated that Cx43 is present in exosomes isolated from biological fluids, namely coronary perfusates from rat hearts in a Langendorff apparatus, culture medium of organotypic heart slices and human plasma (Fig. 3), thus suggesting a new biological and physiological role for Cx43 in mediating cell-exosome communication.

### Cx43 is present at the membrane of exosomes in the form of hexameric channels

**Figure 4:**
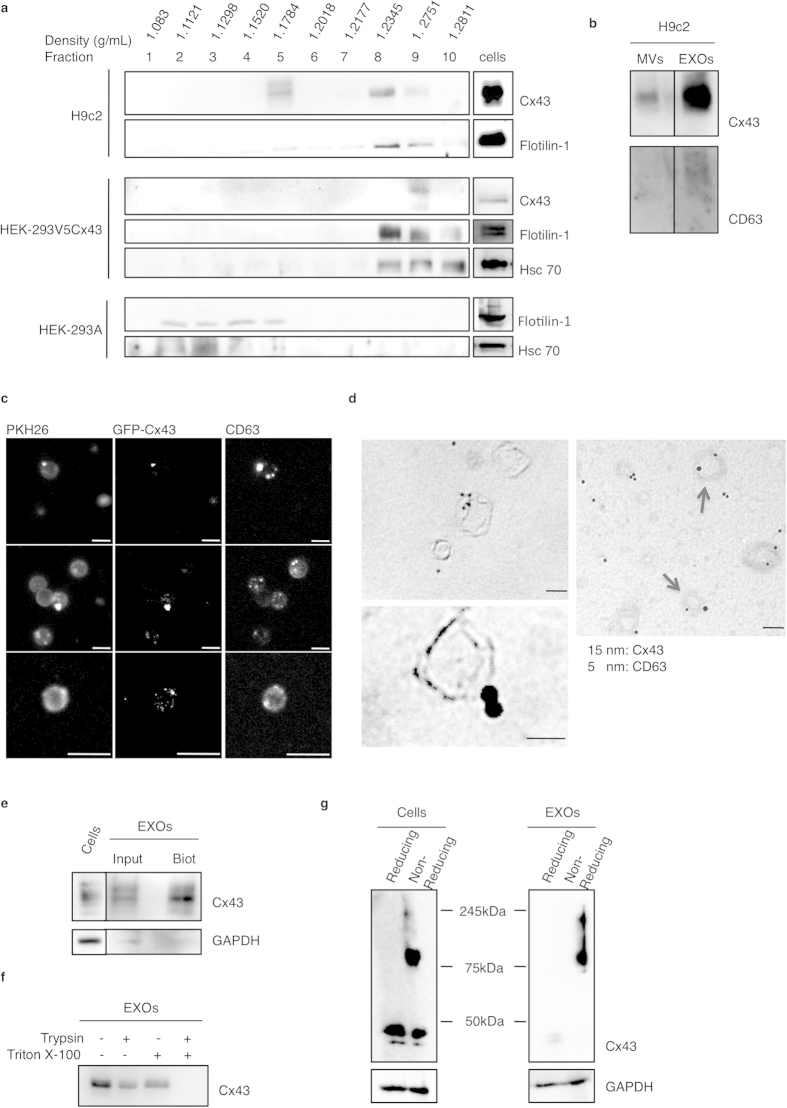
Cx43 is present at the membrane of exosomes forming hexameric channels. (**a**) Exosome flotation in sucrose gradients was performed in exosomes isolated from H9c2, HEK-293V5Cx43 and HEK-293A cells. 15 μg of total protein in exosomal pellets was used in each experiment. The presence of Cx43, Flotillin-1 and Hsc 70 was evaluated by WB. The density of each fraction was determined with a refractometer. (**b**) Microvesicles (MVs) and exosomes (EXOs) were isolated from H9c2 cells. MVs were collected from the 16,500 *g* pellet, whereas exosomes were collected from the 120,000 *g* pellet. Cx43 levels were assessed by WB. (**c**) Exosomes isolated from HEK-293GFPCx43 were labelled with PKH26, attached to 4 μm latex beads and immunostained against CD63 (MX-49.129.5). Images were analyzed by fluorescence microscopy. Scale bars 5 μm. All panels correspond to the same experiment capturing different beads with different magnifications. (**d**) Immunogold and TEM were performed in exosomes isolated from H9c2 (left panel) and HEK-293V5Cx43 cells (right panel). Cx43 (H-150) was labelled with secondary antibodies conjugated with 15 nm gold particles and CD63 (MX-49.129.5) with 5 nm gold particles. Scale bars 100 nm. (**e**) 20 μg EXOs isolated from HEK-293V5Cx43 were biotinylated and precipitated with Neutravidin. Precipitates were eluted in Laemmli buffer and analyzed by WB. (**f**) Trypsin resistance assay was performed in exosomes (5 μg exosomes/condition) isolated from H9c2 cells, either treated or not with 1% Triton X-100. Cx43 levels were assessed by WB. (**g**) Exosome pellets (5 μg/condition) isolated from HEK-293V5Cx43 and cell extracts were lysed in Laemmli buffer or in a non-reducing loading buffer, followed by WB analysis.

To establish that Cx43 is enriched in exosomes rather than in other types of EVs, we performed a sucrose gradient purification to evaluate whether Cx43 is present in fractions with characteristic exosomal densities (Fig. 4a). We demonstrated that, in exosomes isolated from H9c2 cells, Cx43 is found in the same fractions as Flotillin-1, a membrane-associated protein enriched in exosomes. However, both Cx43 and Flotillin-1 can be detected in two fractions, being one of them above the classical range of densities previously described for exosomes. This suggests that our exosomal-enriched pellet might contain MVs, or, alternatively, that the presence of Cx43 induces a shift in the density of the Cx43-enriched isolated fractions. By differential ultracentrifugation, which allows the separation of exosomes and MVs, we showed that the levels of Cx43 in MVs are much lower when compared to exosomes (Fig. 4b). In addition, when we performed a sucrose gradient flotation of exosomes from HEK-293A and HEK-293V5Cx43 (Fig. 4a), we observed that exosomes from the cell line overexpressing Cx43 have the same flotation pattern of H9c2 exosomes, with Cx43, Hsc70 and Flotillin-1 being mainly recovered between 1.21–1.28 g/mL fractions, whereas in HEK-293A, Flotillin-1 and Hsc70 are recovered in fractions ranging from 1.12 and 1.20 g/mL, the densities where exosomes are commonly found. This data suggests that the presence of Cx43 modifies the flotation profile of exosomes, inducing a delayed flotation capacity of the vesicles. A similar effect has been previously reported for the tetraspanin CD63^18^.

In an attempt to evaluate whether Cx43 is present in the same vesicles as CD63, we performed confocal microscopy with exosomes isolated from HEK-293 cells overexpressing GFP-Cx43, labelled with PKH26 and probed with antibodies against CD63. Since exosomes are too small to be analyzed by light microscopy, we first attached the exosomes to latex beads. The colocalization of these 3 markers in discrete points, at the surface of latex beads, indicates the presence of exosomes positive for Cx43 and CD63 (Fig. 4c). Furthermore, we used immunogold labelling and transmission electron microscopy (TEM), to investigate the distribution of Cx43 in exosomes. Strikingly, using exosomes isolated from either HEK-293V5Cx43 or H9c2 cells, we demonstrated that Cx43 is localized at the exosomal membrane, and partially resides in the same vesicles containing CD63 (Fig. 4d). Similar to what was observed by confocal microscopy, a heterogeneous population of exosomes can be found, with vesicles labelled either with antibodies against Cx43, CD63 or both, reinforcing the idea of a regulatory sorting mechanism determining the protein content of exosomes.

The presence of Cx43 at the exosome surface was confirmed by biotinylation and trypsin resistance assays, either in the presence or absence of 1% Triton X-100. We observed that exosomal Cx43 can be biotinylated (Fig. 4e) and that trypsin treatment leads to a decrease of about 60% in Cx43 levels, demonstrating that the protein is being cleaved on the extracellular loops. The total degradation of the protein in the presence of Triton X-100 is likely due to the permeabilization of the exosome membrane, which enables trypsin to access the intracellular domains of Cx43 and fully digest the protein (Fig. 4f). Importantly, when the SDS-PAGE is performed on a 15% polyacrylamide gel it is possible to observe bands of lower molecular weight that can be ascribed to Cx43 fragments (Supplementary Fig. 3a). Altogether, these results demonstrate that Cx43 is embedded in the exosomal membrane, most likely in the form of a channel. Since in cells, Cx43 oligomerizes to form hexameric structures, we proceeded to evaluate whether the same occurs with exosomal Cx43, by performing WB in non-reducing conditions, to preserve oligomer assembly. Figure 4g shows that the electrophoretic migration profile of exosomal Cx43 is similar to that of cells, both in reducing and non-reducing conditions. In both cases, in non-reducing conditions, Cx43 is detected at higher molecular weights, strongly suggesting that in exosomes, Cx43 is also present in the form of oligomers. Lastly, we investigated the transmembrane orientation of Cx43 on the exosomal membrane, using two different approaches. First, we immunoprecipitated Cx43 with antibodies against the C-terminal of the protein, theoretically localized intraluminally, either in the presence or absence of permeabilizing agents (Supplementary Fig. 3b). Cx43 was only immunoprecipitated in the presence of permeabilizing agents. Strikingly, when we used a Cx43 construct with an HA tag in the extracellular loop, we could immunoprecipitate Cx43, with antibodies against HA, even in the absence of detergents (Supplementary Fig. 3c). Altogether, these results demonstrate that Cx43 orientation is the same on the membranes of exosomes and cells.

The results presented in Fig. 4b–g were consistently reproduced in various cell lines, including those endogenously expressing Cx43, such as ARPE or H9c2 cells, and others stably overexpressing GFP-Cx43 or V5-Cx43 (data not shown).

### Exosomal Cx43 facilitates the delivery of heterologous DNA and exosomal uptake by target cells

**Figure 5:**
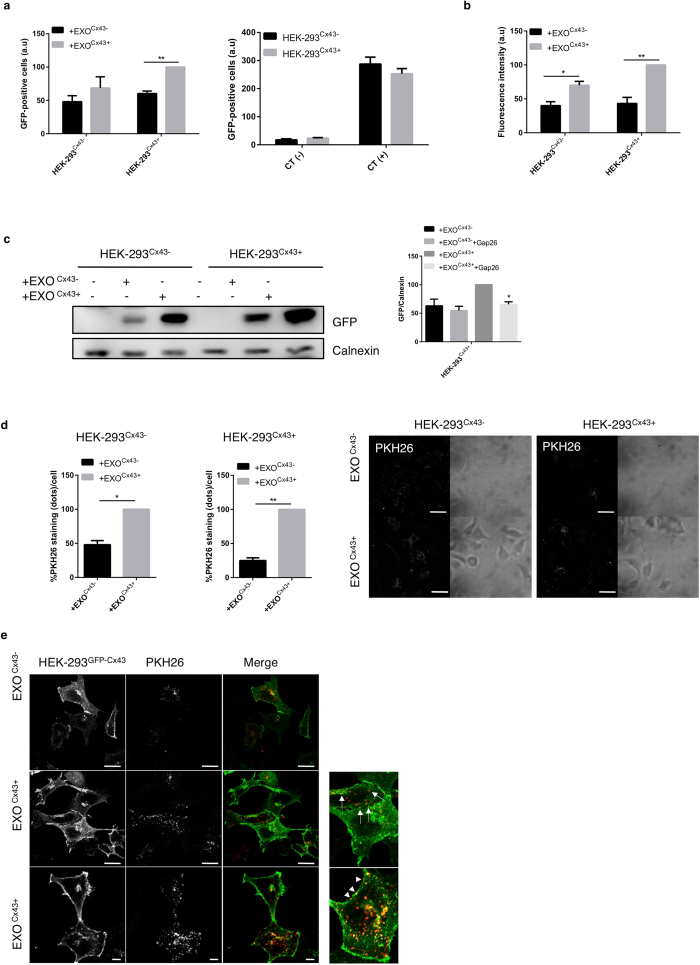
Exosomal Cx43 facilitates the delivery of heterologous DNA and exosomal uptake by target cells. (**a**) Equal amounts of Exo^Cx43−^ and Exo^Cx43+^ were loaded with DNA encoding for GFP using Lipofectamine. Exosomes were subsequently added to recipient cells, HEK-293^Cx43−^ or HEK-293^Cx43+^. Untreated cells were used as negative controls, whereas positive controls were generated by standard transfection procedures. Representative graph shows the flow cytometry analysis of cells incubated with DNA-loaded exosomes. Values represent the count of GFP-positive cells (n = 3), **p < 0.01. (**b**) Representative graph shows the fluorescence microscopy analysis of cells incubated with DNA-loaded exosomes. Values represent the count of GFP-positive cells (n = 3), *p < 0.05, **p < 0.01. (**c**) Representative WB shows the levels of GFP expression in cells incubated with DNA-loaded exosomes. Untreated cells were used as negative controls. Values represent the levels of GFP expression (n = 3), *p < 0.05. (**d**) Equal amounts of Exo^Cx43−^ and Exo^Cx43+^ were labelled with PKH26, purified by flotation in a sucrose gradient and then added to HEK-293^Cx43−^ or HEK-293^Cx43+^ cells. Representative graph and figures show the confocal microscopy analysis. Scale bars: 20 μm. Values represent the count of PKH26 dots/cell (n = 3), *p < 0.05, **p < 0.01. (**e**) Equal amounts of Exo^Cx43−^ and Exo^Cx43+^ labelled with PKH26 were added to recipient HEK-293^GFPCx43^ cells. Representative figures of confocal microscopy analysis. Middle and lower panels are replicates of the same condition, with different magnifications. Scale bars 20 μm. Arrows indicate colocalization of PKH26-stained vesicles with Cx43 inside the cell, and arrow heads point to colocalization at the plasma membrane.

To address the role of Cx43 in vesicle uptake, we loaded exosomes, containing Cx43 (Exo^Cx43+^) or not (Exo^Cx43−^), with a plasmid encoding for GFP. These vesicles were then added to cells, expressing or not Cx43, after which we determined the levels of GFP by flow cytometry, WB or fluorescence microscopy. Consistently, and regardless of the approach used to evaluate the expression of GFP in target cells, the highest value was invariably observed when Cx43 is present both in exosomes and acceptor cells (Fig. 5a–c). Altogether, these results suggest that Cx43 can mediate the transfer of plasmid DNA from exosomes to cells. However, due to size and shape constrains, it is very unlikely that a plasmid can pass through a Cx43 channel pore. Therefore the increased expression of GFP when Cx43 is present both in cells and exosomes may reflect the involvement of Cx43 in facilitating the transfer of the plasmid through a fusion and/or internalization event. To confirm the involvement of Cx43 in exosome internalization, exosomes were either loaded with GFP plasmids or labelled with Acridine Orange (AO) for RNA tracing and subsequently added to cells either in the presence or absence of endocytic inhibitors, such as dynasore, cytochalasin D, and methyl-β-cyclodextrin. In any of these conditions, inhibition of endocytosis resulted in a significant decrease of GFP expression and AO uptake, demonstrating that the internalization of exosomes is being hampered (Supplementary Fig. 4c,d).

Additionally, we stained exosomes with the lipid probe PKH26, before their incubation with cells for 30 min (Fig. 5d). The number of PKH26 (red dots per cell) was used as an indication of the amount of exosomes that enters the cell. Surprisingly, in absolute levels, HEK-293^Cx43+^ cells present about 4 times less red dots than HEK-293^Cx43−^ cells. However, and consistent with previous data, the presence of Cx43 in exosomes resulted in more dots per cell, being this increase around 2 fold in HEK-293^Cx43−^ whereas in HEK-293^Cx43+^ cells the number of red dots per cells increases around 4 fold. Furthermore, when we used HEK-293GFPCx43 as acceptor cells (Fig. 5e), we not only observed an increased number of internalized vesicles when Cx43 is present in exosomes, but also, and more important, we showed an extensive colocalization between red and green dots, suggesting that exosomes are localized in Cx43-containing compartments. Moreover, this colocalization occurs predominantly at the cell periphery, where no adjacent cells are present, or at the apical membrane (lower panel). The absence of PKH26 staining at GJ plaques (staining in cell-cell contacts) strongly indicates that exosomes do not interact with Cx43 when it is forming GJ.

### Cx43 hemichannels present at the exosomal membrane mediate the transfer of exosome content into target cells

**Figure 6:**
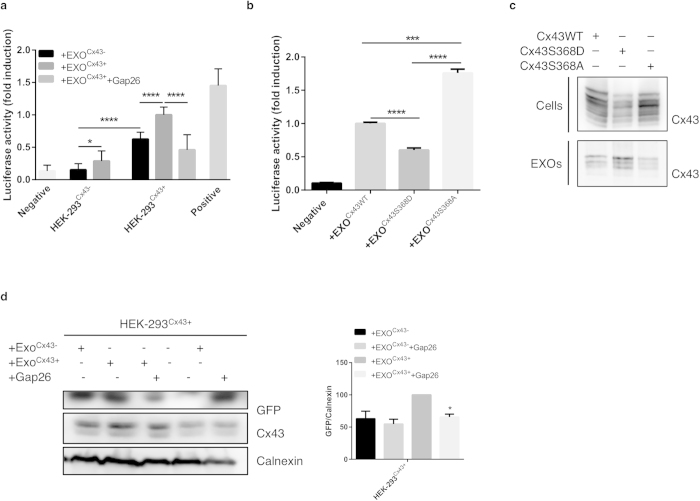
Cx43 hemichannels at the exosomal membrane mediate the transference of exosomal contents into target cells. (**a**) Equal amounts of Exo^Cx43−^ and Exo^Cx43+^ were loaded with luciferin and treated with 0.25 mg/ml gap26 for 30 min, where indicated. Exosomes were added to HEK-293^Cx43−^ or HEK-293^Cx43+^ cells expressing luciferase. Light emission was determined and values were expressed as fold induction over HEK-293^Cx43+^ stimulated with Exo^Cx43+^ exosomes (n = 3) *p < 0.05, ****p < 0.0001. (**b**) Equal amounts of Exo^Cx43WT^, Exo^Cx43S368D^ and Exo^Cx43S368A^ were loaded with luciferin and added to HEK-293^Cx43+^ cells expressing luciferase. Light emission was determined and values were expressed as fold induction over HEK-293^Cx43+^ stimulated with Exo^Cx43WT^ exosomes (n = 3) ***p < 0.001, ****p < 0.0001. (**c**) WB analysis of exosomes (EXOs) isolated from HEK-293^Cx43−^ cells, transfected with Cx43WT, Cx43S368D and Cx43S368A. (**d**) Equal amounts of Exo^Cx43−^ and Exo^Cx43+^ were loaded with DNA encoding for GFP, and treated with gap26 for 30 min, where indicated. Exosomes were subsequently added to HEK-293^Cx43+^ cells. Representative WB shows the levels of GFP expression after 24 h, in cells incubated with DNA-loaded exosomes. Graph depicts GFP expression levels (n = 3), *p < 0.05.

One of the most appealing hypothesis for the role of Cx43 in mediating the communication between exosomes and cells is allowing for the direct transfer of cargo through Cx43-containing channels formed by the docking of exosomes and target cells. To test this hypothesis, we used the luciferin-luciferase system, in which exosomes (Exo^Cx43+^ or Exo^Cx43−^) loaded with luciferin were added to luciferase-expressing cells (either HEK-293^Cx43+^ or HEK-293^Cx43−^). Luciferin has a molecular weight inferior to 1 kDa and is likely to be permeable to GJ channels. The amount of light emitted by the acceptor cell was correlated with the efficiency of luciferin transfer from exosomes to cells. In agreement with our previous data, Fig. 6a shows that when acceptor cells are devoid of Cx43, the presence of Cx43 in exosomes results in a small, but statistically significant increase in the amount of light emitted by luciferase-expressing cells, in comparison with exosomes lacking Cx43. Importantly, when acceptor cells express Cx43, Exo^Cx43+^ led to an increase of about 40% in the levels of luminescence. Since light emission in our experiments occurs a few seconds after the addition of exosomes, a period that is not compatible with exosome internalization, these results indicate that the content of exosomes is being released into acceptor cells through the channel pore independently of vesicle internalization. To determine whether a functional channel is required to mediate such communication we used a mimetic peptide, gap26, previously shown to block hemichannels and inhibit GJ intercellular communication. Strikingly, incubation of Exo^Cx43+^ with gap26, prior to their addition to HEK-293^Cx43+^ cells, results in a 55% inhibition of light emission, suggesting that the transfer of luciferin is hampered, likely due to channel blockage. To further confirm the requirement of functional channels to permit the passage of luciferin, we used exosomes containing Cx43 mutants in the Serine368 residue, that form constitutively open (Cx43S368A) channels^19^. Given that phosphorylation of residue S368 leads to a decrease in intercellular communication, we used a Cx43 mutant (Cx43S368D) that mimics a constitutively phosphorylated form of Cx43, which is likely in a closed state. Consistent with the data presented above, the amount of luciferin that reaches luciferase-expressing cells is higher in Cx43S368A exosomes, strongly supporting that Cx43 channels mediate the passage of exosomal content into cells (Fig. 6b). Data showing that neither the amount of luciferin loaded into exosomes, nor the levels of mutated forms of Cx43 present in the vesicles vary significantly, suggests that the observed effects can be attributed to changes in channel activity (Fig. 6c and Supplementary Fig. 4e).

Furthermore, to evaluate whether gap26 also interferes with the docking of exosomes with target cells we incubated Exo^Cx43+^ loaded with DNA encoding for GFP with gap26 before they were added to HEK-293^Cx43+^ cells. Similar to the luciferin-luciferase system, incubations of cells with gap26 also led to a reduction of about 45% in the levels of GFP expression, suggesting that the mimetic peptide can restrain exosomal docking (Fig. 6d). Importantly, when exosomes are devoid of Cx43, gap26 does not present any effect.

## Discussion

Although several proteins have been associated with cell-exosome coupling, the mechanisms underlying such processes are far from being understood. In this study, using a step-by-step approach, we identify, for the first time, the presence of the GJ protein Cx43 in EVs, including exosomes. Moreover, we demonstrate that Cx43 exists not only in exosomes released by cells in culture, but also in biological fluids, namely in human blood and rat heart perfusates. Furthermore, we show that Cx43 is embedded in the exosomal membrane, in the form of a hexameric channel, suggesting a biological role for exosomal Cx43 channels. Accordingly, our data shows that Cx43 facilitates the interaction between exosomes and cells and mediates the transfer of exosomal cargo into target cells.

The underlying mechanisms that determine the transfer of biological information between exosomes and acceptor cells are still obscure and constitute a matter of intense research. Although some argue that exosomes release their cargo into cells by fusing with the plasma membrane, the prevailing theory suggests that cells internalize exosomes^20^. Most of the experimental evidence gathered thus far indicate the involvement of virtually every known internalization pathway in the uptake of exosomes^20^, suggesting that these vesicles can follow more than one entrance route on their way to the intracellular milieu. Although it was initially thought that exosomal capture and uptake occurs fortuitously, recent studies have demonstrated that this process can be more complex, specific and regulated than previously anticipated. Indeed, reports have consistently shown that prior to fusion and/or internalization, exosomes dock with cells through adhesion molecules, such as the tetraspanins CD9, CD63 and CD81^8^,^21^,^22^,^23^,^24^,^25^, integrins CD51 and CD61^10^ or membrane receptors like galectin-5^26^. This suggests that the presence of certain proteins decorating the cell surface can offer specificity in the process of exosomal targeting to cells. Strikingly, our work now shows that beyond direct GJ-mediated intercellular communication, a novel role can be attributed to the membrane protein Cx43 in mediating the transfer of exosomal content into acceptor cells.

Cxs are transmembrane proteins that oligomerize to form hexameric channels, called hemichannels. When two hemichannels, localized in neighbour cells dock with each other, small substances, such as ions, second messengers, metabolites, mRNA or miRNA can be directly exchanged between the cytoplasm of the two cells^11^,^12^. In the present study, we show that Cx43 also mediates the transfer of biological information between exosomes and cells. The increased expression of the GFP-encoding plasmid, loaded into exosomes, suggests that Cx43 is somehow mediating the transfer of the plasmid from exosomes to cells. However, due to size and shape constrains, it is very unlikely that a plasmid can pass through a Cx43 channel pore. Even though the incubation period of cells with exosomes loaded with GFP plasmids spanned for only 30 minutes to partially restrain endocytosis, the increased expression of GFP in these circumstances may reflect the involvement of Cx43 in facilitating the transfer of the plasmid through a fusion and/or internalization event. This hypothesis is supported by our data demonstrating that incubation of exosomes with the mimetic peptide gap26, which is capable of blocking Cx43-containing channel activity, partially inhibits the expression of GFP in acceptor cells, presumably by restraining exosomal docking. Moreover, using PKH26-labelled exosomes, we show that the presence of Cx43 in exosomes increases the amount of exosomal vesicles detected in target cells, an effect particularly evident when the acceptor cells also express Cx43. Surprisingly, we found that if the incubation of exosomes is carried out in cells devoid of Cx43, the absolute levels of PKH26 dots per cell is significantly higher when compared with Cx43-expressing cells. One of the major caveats of using fluorescent lipid membrane dyes is the possibility of dye leaching into cellular membranes when labelled exosomes reach the cell^20^. Therefore, it is conceivable that lipid dye transfer can lead to a dilution effect with the consequent detectable signal loss by fluorescence acquisition techniques. In this context, it is reasonable to speculate that an augmented docking and/or fusion of exosomes with the plasma membrane, due to the presence of Cx43 at the cell surface, may result in a faster dilution of the dye within the cellular lipid environment. Consequently, this effect would ultimately lead to a decrease, instead of an increase, of the absolute PKH26 fluorescence found in cells. Moreover, it is also plausible that the presence of Cx43 at the plasma membrane modifies the surrounding lipid environment with the consequent alteration of membrane biophysical properties, which can subsequently affect the interaction of exosomes with acceptor cells. Interestingly, our data shows that the presence of Cx43 in exosomes already modifies the flotation profile of these vesicles. Indeed, Cx43 induces a delayed flotation capacity on exosomes, as demonstrated by a shift of Flotillin-1 flotation to higher densities in sucrose gradients. However, this is not without precedent, since it has been reported that an increase in the amount of another membrane protein in exosomes, the tetraspanin CD63, had a similar effect^18^.

In this study, we presented data showing that Cx43 modulates the targeting and/or docking of exosomes with acceptor cells. However, several other membrane proteins have been described to mediate the interplay between cells and exosomes, which may simply imply that there is redundancy between the different molecular players. Nevertheless, since the absence of Cx43 has such a significant impact on the transfer of exosomal cargo to target cells, we propose that Cx43 plays a central role in the specificity and robustness of the mechanism. Moreover, it is also plausible that a population of exosomes, particularly enriched in Cx43, has a different biological role, when compared to Cx43-depleted exosomes. Indeed, our data indicates that proteins can be differentially loaded into exosomes, with some vesicles containing both Cx43 and CD63, while others are exclusive for only one of the proteins, suggesting the existence of a mechanism that regulates which proteins are sorted into exosomes during their biogenesis.

Our experiments performed using exosomes loaded with luciferin, a dye with a molecular weight (9240 Da) that is compatible with the size range of substances that can pass through GJ, provides strong evidence that, besides a role in exosomal docking, Cx43 channels might also mediate the transfer of molecules between exosomes and cells without prior internalization of the vesicle. This hypothesis is strongly supported by data showing that gap26 significantly reduces the amount of light emitted by luciferase-expressing cells, suggesting that the access of luciferin to the cytoplasm of target cells is hindered by the channel blocker. Furthermore, using Cx43 mutants that form constitutively open or closed channels, we demonstrated that a functional pore is required to allow the passage of exosomal content into the recipient cell. Since light emission occurs a few seconds after the addition of the exosomes, a period that is not compatible with exosomal internalization, these results indicate that the content of exosomes can be released into acceptor cells independently of vesicle internalization, through the Cx43 channel pore. By analogy with other membrane proteins, it is conceivable that the Cx43 present in exosomes derives from the endocytic pathway, and is incorporated into exosomes during the inward budding of MVBs, thus, we envision a mechanism whereby internalized Cx43 can be diverted from degradation and reused as hemichannels, to mediate exosome-cell communication. Overall we propose a model where Cx43 facilitates the docking of exosomes to target cells, through a process that may lead to the formation of a GJ-like structure, capable of transferring some of the exosomal cargo into the cell, directly through the channel pore. Our hypothesis also supports a model where signal dissemination between cells through the action of Cx43-loaded exosomes might be much faster than anticipated. Therefore, the results described in this study extend the functional scope of Cx43 from GJ-mediated intercellular communication between neighbour cells to GJ communication between cells at potentially much longer distances and even between different tissues and organs.

## Material and Methods

Additional information is appended in Supplementary Materials and Methods.

### Animal models

Wistar rats were handled according to EU guidelines (86/609/EEC), with approval of the Ethics Committee, Faculty of Medicine, University of Coimbra, Portugal. For Langendorff experiments, hearts were isolated from 12-week-old rats, perfused for 30 min with modified Krebs-Henseleit (KH) buffer (in mM: 118 NaCl, 25 NaHCO3, 4.7 KCl, 1.2 MgSO4, 1.2 KH2P04, 10 HEPES, 1.25 CaCl2 and 10 glucose, pH 7.49), equilibrated with 95% O_2_/5% CO_2_ at 37 °C, as previously described^27^. For organotypic cultures, hearts from post-natal day 7 rats were excised and ventricles sagitally sliced with a rodent heart matrix (Harvard Apparatus). Heart slices were transferred to a semiporous membrane (Millicel-CM 0.4 mm, Millipore), and placed in 6-well plates with exosome-depleted medium [1 mL DMEM with 2 mM GlutaMAX, 10% FBS and Penicillin/Streptomycin (100 U/mL:100 μg/mL)], for 48 h, in a humidified atmosphere containing 5% CO_2_, at 37 °C^28^.

### Exosome purification

Cells were cultured in exosome-depleted medium, prepared accordingly to Lässer *et al.*^29^. After incubation for 24 or 48 h, as indicated, medium was collected and exosomes were isolated by ultracentrifugation, as previously described^29^. Harvested supernatants were subjected to differential centrifugation at 4 °C, starting with a 10 min centrifugation at 300 *g*, followed by 20 min at 16,500 *g*. To thoroughly remove cellular debris and larger particles, supernatants were filtered through a 0.22-μm filter unit, followed by ultracentrifugation at 120,000 *g*, for 70 min. The resultant pellet was washed with PBS, and after ultracentrifugation, exosomes were resuspended in PBS. On average, for the cell lines used in this study, 10 μg of purified exosomes were obtained from 20 million cells. The same method was applied for exosomal purification from organotypic heart slices and heart perfusates. Human blood samples were withdrawn from healthy volunteers and exosome isolation was performed as previously described^30^. 3 ml plasma were diluted in PBS and centrifuged 30 min at 2,000 *g*, at 4 °C. Supernatants were ultracentrifuged for 45 min at 12,000 *g*, followed by 2 h at 110,000 *g*. Pellets were resuspended, diluted in PBS and filtered through a 0.22-μm filter. Subsequently, two ultracentrifugations of 110,000 *g* for 70 min, were performed.

### Nanosight tracking analysis (NTA)

Exosomes isolated from H9c2 cells were subjected to Nanosight tracking analysis (NTA), using a NanoSight LM 10 instrument (NanoSight Ltd). Settings were optimised and kept constant between samples. Each video was analyzed to give mean, mode, median and estimated concentration for each particle size^31^. Data were processed using NTA 2.2 analytical software.

### Targeted SWATH for detection of Cx43 in exosomes

A specific library of precursor masses and fragment ions for Cx43 was created. Briefly, a sample enriched in Cx43 (an immunopurification sample of Cx43 with a V5 tag – HEK-293V5Cx43) was used in Information Dependent Acquisition (IDA) experiments.

### Separation of vesicles on sucrose gradient

Exosome-enriched pellets were placed at the bottom of an ultracentrifuge tube, filled with a discontinuous gradient of sucrose (from 2.5 M to 0.4 M) and ultracentrifuged overnight at 160,000 *g*. Sequential fractions were collected (1 mL each, from the top to the bottom of the tube), and densities were determined using an Atago Uricon-N Hand-held Refractometer. Fractions were washed with PBS by ultracentrifugation for 70 min, at 120,000 g^32^ and further denatured and analyzed by WB.

### Immunofluorescence staining of exosomes

5 μg of HEK-293GFPCx43 purified exosomes were incubated with surfactant-free 4 μm aldehyde/sulphate latex beads (Life Technologies) for 2 h at room temperature (RT), under agitation. Blocking was performed with 100 mM of glycine, for 30 min. Beads were washed with PBS/0.5% BSA, followed by incubation with anti-CD63 antibodies, for 30 min at 4 °C, and incubation with secondary antibodies for 30 min. Samples were rinsed with PBS. 1 μL exosomes was layed in a cover slip using a hydrophobic barrier pen and mounted with MOWIOL 4–88 Reagent. Images were collected in a confocal microscope, Zeiss LSM 710 (Carl Zeiss).

### Transmission electron microscopy (TEM) and immunogold

Exosomes were fixed with 2% paraformaldehyde (PFA) and deposited on Formvar-carbon coated grids (TAAB Laboratories). Samples were washed with PBS and fixed with 1% glutaraldehyde for 5 min. After washing with distilled water, grids were contrasted with uranyl-oxalate pH 7, for 5 min, and transferred to methyl-cellulose-uranyl acetate, for 10 min on ice^30^. For immunogold staining, exosomes were adsorbed on Formvar-carbon coated grids, permeabilized with 0.1% saponin, washed and blocked with 0.5% BSA. Exosomes were incubated with rabbit anti-Cx43 (H-150) and mouse anti-CD63 (MX-49.129.5), washed and labelled with secondary antibodies conjugated to gold particles. Grids were contrasted as described above. Observations were carried out using a Tecnai G2 Spirit BioTWIN electron microscope (FEI) at 80 kV.

### Biotinylation of exosome-surface proteins

20 μg exosomes, from HEK-293V5Cx43 cells, were incubated with 0.5 mg/ml Sulfo-NHS-SS-biotin in PBS containing 0.5 mM MgCl_2_ and 1 mM CaCl_2_, for 30 min, and washed with PBS/Ca^2+^/Mg^2+^ by ultracentrifugation. Exosome pellets were lysed in RIPA buffer and Neutravidin beads were added for 2 h, at 4 °C. Beads were pelleted and precipitates were eluted in Laemmli buffer and denatured, followed by WB analysis (see Supplementary Materials and Methods).

### Trypsin resistance assay

After exosome isolation, Trypsin resistance assays were performed using 5 μg exosomes/condition. Trypsin was used to remove peripherally associated proteins, either in the presence or absence of 1% Triton X-100. 1 mg/mL of Trypsin was added for 10 min at 37 °C, and further inactivated with 1 mg/mL soybean Trypsin inhibitor. Exosomes were denatured with Laemmli buffer and analyzed by WB.

### Chemical loading of exosomes

For chemical loading of exosomes, Exo^Cx43−^ or Exo^Cx43+^, were obtained from HEK-293A (HEK-293^Cx43−^) or HEK-293V5Cx43 (HEK-293^Cx43+^), respectively. 5 μg plasmid DNA encoding for GFP was mixed with 10 μg Lipofectamine 2000 (Life Tecnhologies) in Optimem for 10 min, at RT^33^. 25 μg exosome suspension was added and the mixture incubated for 30 min, at RT. To eliminate DNA present outside of the exosomes, complexes were incubated with 5 U DNAse I (Takara Bio), for 30 min, at 37 °C. The mixture was purified by washing with PBS and ultra-filtration through a 100-kDa filter (Amicon, Millipore), 3 times, in order to eliminate Lipofectamine-loaded with GFP, DNase and its degradation products^34^. To attest the efficiency of the purification procedure, Lipofectamine-DNA miceles were incubated with DNase I and further ultra-filtrated through a 100-kDa filter. Filtrates were either co-cultured with recipient cells or subjected to agarose gel electrophoresis of DNA to verify DNA integrity (Supplementary Data 4).

Recipient HEK-293^Cx43−^ or HEK-293^Cx43+^ cells were cultured in 24-well plates. To specifically inhibit Cx43 hemichannels, we pre-treated the exosomes with a synthetic mimetic peptide, derived from the first extracellular loop of Cx43, gap26 (0.25 mg/ml), for 30 min, at 37 °C^35^. 2.5 μg exosomes loaded with DNA were co-cultured with the recipient cells, for 30 min, for transfection with heterologous DNA. After that, culture medium was replaced with exosome-depleted medium, and left for 24 h of protein expression. GFP expression in recipient cells was evaluated by flow cytometry, fluorescence microscopy or WB. For flow cytometry analysis, cells were detached with Trypsin, centrifuged and resuspended in DMEM (low glucose, without phenol red). Flow cytometry was performed in a FACS Calibur flow cytometer (BD Biosciences) and analyzed using Cell Quest software. For fluorescence microscopy imaging, cells were fixed in 4% PFA. Specimens were mounted with MOWIOL before image collection, using a Zeiss LSM 710.

### Exosomal PKH26 dye uptake

For dye uptake assays, 5 μg of Exo^Cx43−^ or Exo^Cx43+^ were labelled with PKH26 Fluorescent Cell Linker, resuspended in Diluent C, for 5 min. Excess dye was washed by exosome flotation on a sucrose gradient. 4–10 fractions were collected and washed with PBS by ultracentrifugation. 2.5 μg exosomes were co-cultured with recipient cells, grown on glass coverslips, for 30 min. Cells were fixed with 4% PFA. Specimens were mounted with MOWIOL before image collection, using a Zeiss LSM 710. Nuclei were stained with DAPI. Quantification of internalized exosomes was performed using Image J.

### Exosomal Luciferase assay

HEK-293^Cx43−^ or HEK-293^Cx43+^ cells were seeded in a white 96-well clear-bottom plate, and transfected 24 h after seeding with luciferase encoding plasmid, using Lipofectamine 2000. After transfection, medium was replaced by exosome-depleted medium, without phenol red and cells were allowed to grow for 24 h. Exo^Cx43−^ or Exo^Cx43+^ were loaded with DMNPE-caged-luciferin for 1 h, at 37 °C, protected from the light. Luciferin was released from the DMNPE group by UV-B (365 nm) photolysis (5 min, on ice). Where indicated, Exo^Cx43+^ were pre-treated with gap26 (0.25 mg/ml) for 30 min, at 37 °C. Non-incorporated luciferin and gap26 were removed from the exosome suspension using Exosome Spin Columns (Life Technologies). The reaction was initiated by adding non-treated exosomes, luciferin-loaded exosomes, or luciferin to the recipient cells. Luciferase activity was determined and data points were recorded using a Biotek Synergy HT microplate reader with Gen 5 software (Biotek).

### Statistical analysis

All data are representative of at least three independent experiments. Data were analyzed with GraphPad Prism 6 for Windows, version 6.01. Unless stated otherwise, values depicted on graphs are expressed as mean ± SEM. As appropriate, Student’s t-test with Welch correction or ANOVA, followed by Kruskal-Wallis multiple comparison tests were applied. Differences were considered significant at P < 0.05.

## Additional Information

**How to cite this article**: Soares, A. R. *et al.* Gap junctional protein Cx43 is involved in the communication between extracellular vesicles and mammalian cells. *Sci. Rep.* **5**, 13243; doi: 10.1038/srep13243 (2015).

## Supplementary Material

Supplementary Information
