# Corrigendum: Gap junctional protein Cx43 is involved in the communication between extracellular vesicles and mammalian cells

**DOI:** 10.1038/srep14888

**Published:** 2015-10-08

**Authors:** Ana Rosa Soares, Tania Martins-Marques, Teresa Ribeiro-Rodrigues, Joao Vasco Ferreira, Steve Catarino, Maria João Pinho, Monica Zuzarte, Sandra Isabel Anjo, Bruno Manadas, Joost P.G. Sluijter, Paulo Pereira, Henrique Girao

Scientific Reports 5: Article number: 13243; 10.1038/srep13243 published online: 08192015; updated: 10082015.

The Acknowledgements section in this Article is incomplete.

“This work was supported by Portuguese Foundation for Science and Technology (FCT) grants PTDC/SAU-ORG/119296/2010, PTDC/SAU-ORG/118694/2010 and PEST-C/SAU/UI3282/2013-COMPETE.”

should read:

“This work was supported by Portuguese Foundation for Science and Technology (FCT) grants PTDC/SAU-ORG/119296/2010, PTDC/SAU-ORG/118694/2010, PEST-C/SAU/UI3282/2013-COMPETE and FCT-UID/NEU/04539/2013.”

