# Supplementary material for: Gap junctional protein Cx43 is involved in the communication between extracellular vesicles and mammalian cells
Source: Sci Rep. 2015 Aug 19;5:13243. doi: 10.1038/srep13243 (PMC4541155; doi:10.1038/srep13243)
Supplement: Supplementary Information [file srep13243-s1.pdf]

Gap junctional protein Cx43 is involved in the communication between extracellular vesicles and mammalian cells

Ana Rosa Soares[1,6], Tania Martins-Marques[1,6], Teresa Ribeiro-Rodrigues[1], Joao Vasco Ferreira[1], Steve Catarino[1], Maria João Pinho[1], Monica Zuzarte[1], Sandra Isabel Anjo[2,3], Bruno Manadas[2], Joost Sluijter[4,5], Paulo Pereira[1], Henrique Girao[1‡]

[1]Centre of Ophthalmology and Vision Sciences, Institute of Biomedical Imaging and Life Sciences (IBILI), Faculty of Medicine, University of Coimbra, Azinhaga de Sta Comba, 3000-354 Coimbra, Portugal

[2]CNC - Center for Neuroscience and Cell Biology, University of Coimbra, 3004-517 Coimbra, Portugal

[3]Department of Life Sciences, Faculty of Sciences and Technology, University of Coimbra, 3004-517 Coimbra, Portugal

[4]Department of Cardiology, Division of Heart & Lungs, University Medical Center Utrecht, the Netherlands

[5] Interuniversity Cardiology Institute Netherlands (ICIN), Utrecht, the Netherlands

[6]Co-first authors

Address: Centre of Ophthalmology and Vision Sciences, Institute of Biomedical Imaging and Life Sciences (IBILI), Faculty of Medicine, University of Coimbra, Azinhaga de Sta Comba, 3000-354 Coimbra, Portugal

‡Corresponding Author: Henrique Girao – hmgirao@fmed.uc.pt

SUPPLEMENTAL TABLES

(a)

Common protein pattern of exosomes			
**Classification**		**Protein**	**Targeted-SWATH-MS**
Antigen presentation		MHC class I	
		MHC classII	
Adhesion molecules	Tetraspanins	CD63	x
		CD81	x (CD81P3)
		CD9	
		CD37	
		CD53	
		CD82	
	Integrins	α3	x
		α4	
		αM	
		αL	
		β1	x
		β2	
Membrane transport and fusion	Annexins	I	x
		II	x
		IV	x
		V	x
		VI	x
		VII	
		XI	
		Flotilin-1	
	Rab	2	
		5c	x
		7	
		10	
	Arf	3	
		5	
		6	
ESCRT proteins		Alix	x
		Tsg 101	
Heat-Shock proteins		Hsc70	x
		Hsp90	x
Cytoskeletal proteins		Actin	x
		Cofilin 1	
		Moesin	x
	Tubulin	α1	x
		α2	
		α6	
		β3	x
		β5	
Enzymes		Pyruvate kinase	x
		Alpha enolase	x
		GAPDH	x
Signal transduction		14-3-3ε	x
		14-3-3γ	x
		14-3-3ζ	x
		Gβ1	x

		Gi2α	x
Histones		H2B	x
		H2A	x
		H4	x
Others		MFG-E8 (lactadherin)	x
		Clathrin	x
		Elongation factor 1α	x
		C3	
		Lamp2	x (1)

(b)

Proteins more often identified in exosomes (by frequency)		
**Protein**		**Targeted-SWATH-MS**
HSC70	heat shock 70kDa protein 8	X
CD9	CD9	
GAPDH	glyceraldehyde-3-phosphate dehydrogenase	X
ACTB	Actin, cytoplasmic 1	X
CD63	CD63	X
CD8A	CD8A	
ANXA2	annexin A2	X
ENO1	enolase 1	X
HSP90A	heat shock protein 90kDa	X
eEF1A1	eukaryotic translation elongation factor 1 alpha 1	X
YWHAE (14-3-3ε)	tyrosine 3-monooxygenase/tryptophan 5-monooxygenase activation protein	X
SDCBP	syntenin	X
PDCD6IP	Alix (programmed cell death 6 interacting protein)	X
ALB	albumin	X
YWHAZ (14-3-3ζ)	tyrosine 3-monooxygenase/tryptophan 5-monooxygenase activation protein	X
eEF-2	eukaryotic translation elongation factor 2	
ACTG1	cytoskeletal gamma-actin	X
LDH-A	lactate dehydrogenase A	X(B)
HSP90A	heat shock protein 90kDa alpha	X
ALDOA	aldolase A, fructose-bisphosphate	X
MSN	moesin	X
ANXA5	annexin A5	X
PGK1	phosphoglycerate kinase 1	
CFL1	cofilin 1	

Table S1 - Detection of exossomal proteins by targeted-SWATH-MS. (a) List of the proteins more commonly found in exosomes. In the right column, exosomal proteins identified by our targeted-SWATH method are highlighted. **(b)** List of the exosomal proteins more commonly identified in previous

proteomic studies. In the right column, exosomal proteins identified by our targeted-SWATH method are highlighted.

<table>
<tr><th></th><th>Peptide Sequence</th><th>Peptide m/z</th><th>RT</th><th>SWATH window (m/z)</th></tr>
<tr><td rowspan="7">Cx43</td><td>YGIEEHGK</td><td>466.7256</td><td>6.2</td><td>464-474</td></tr>
<tr><td>VQAYSTAGGK</td><td>491.2515</td><td>5.83</td><td>483-494</td></tr>
<tr><td>SDPYHATTGPLSPSK</td><td>519.9237</td><td>10.98</td><td>513-524</td></tr>
<tr><td>TYIISILFK</td><td>549.3325</td><td>24.7</td><td>543-554</td></tr>
<tr><td>VAQTDGVNVEM[Oxi]HLK</td><td>778.8876</td><td>11.230</td><td>773-784</td></tr>
<tr><td>M[Oxi]GQAGSTISNSHAQPFDFPDDNQNAK</td><td>931.7444</td><td>14.5269</td><td>923-934</td></tr>
<tr><td>QASEQNWANYSAEQNR</td><td>948.4125</td><td>11.2544</td><td>943-950</td></tr>
<tr><td rowspan="5">iRT peptides</td><td>TGFIIDPGGVIR</td><td>622.8525</td><td>21.0609</td><td>613-624</td></tr>
<tr><td>TPVISGGPYYER</td><td>669.836</td><td>14.4352</td><td>663-674</td></tr>
<tr><td rowspan="2">VEATFGVDESANK</td><td rowspan="2">683.8262</td><td rowspan="2">13.0164</td><td>673-684</td></tr>
<tr><td>683-694</td></tr>
<tr><td>GDLDAASYYAPVR</td><td>699.3371</td><td>16.1109</td><td>693-704</td></tr>
</table>

Table S2 - SWATH windows used for the targeted analysis. For targeted-SWATH analysis for detection of Cx43 in exosomes, a set of 12 windows was constructed covering the precursor mass range of the peptides identified for Cx43 and for the iRT peptides.

SUPPLEMENTARY FIGURES

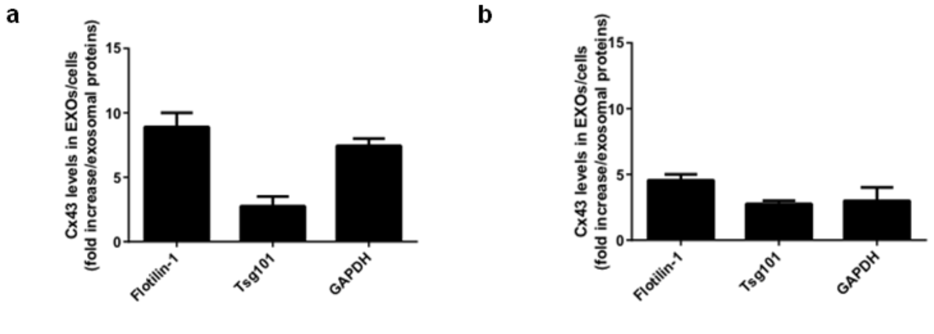

Supplementary Figure 1 – Cx43 is particularly enriched in exosomes secreted by different cell lines. Representative graph show the ratio of the levels of Cx43 in exosomes/cells as fold increase over the levels of other exosomal proteins, in **(a)** HEK-293V5Cx43 cells and **(b)** HEK-293GFPCx43 cells.

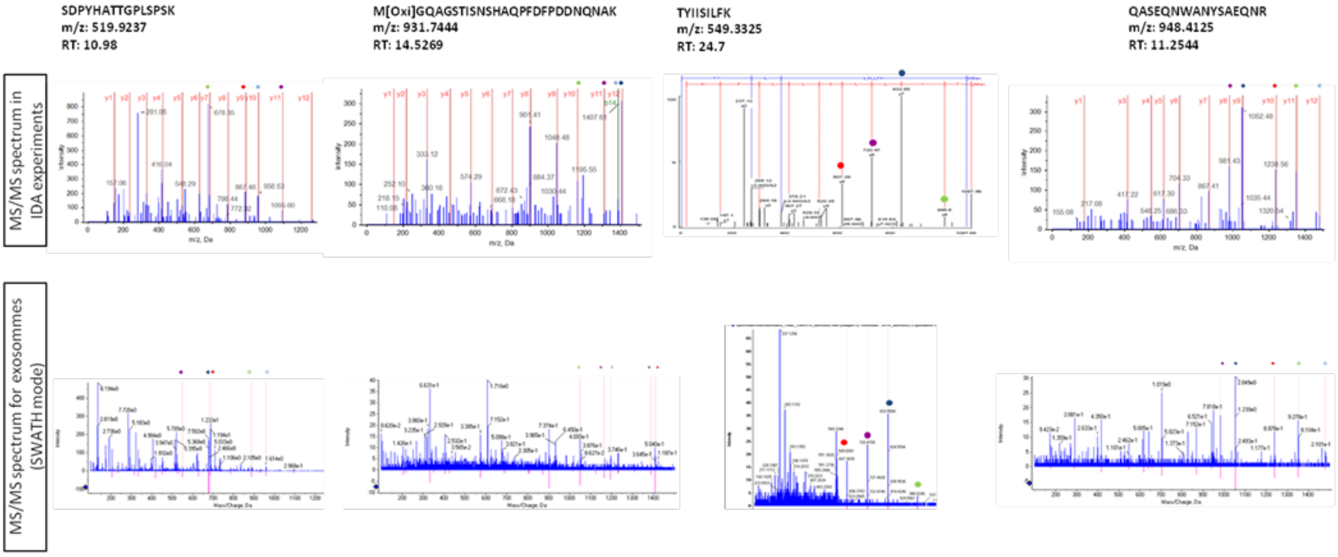

Supplementary Figure 2 – Detection of Cx43 in exosomes by targeted-SWATH-MS. Fragmentation spectra of the identified peptides of Cx43 (top panel) and fragmentation spectra obtained in the analysis of the exosomal sample using the targeted-SWATH method (bottom panel). For each spectrum it is presented the peptide sequence, its m/z and RT values. The transitions used are indicated with colored balls according with its respective extracted ion trace color.

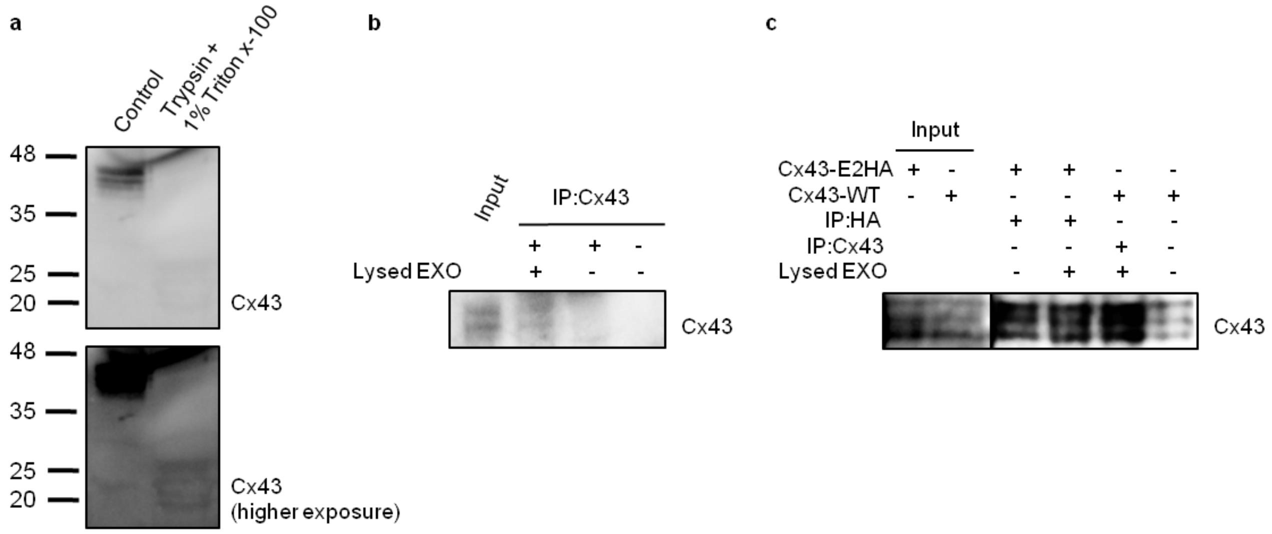

Supplementary Figure 3 – Cx43 presents the same orientation in the membrane of exosomes, as in the plasma membrane of cells. (a) Trypsin resistance assay was performed for 20 min in exosomes (5 μg exosomes/condition) isolated from H9c2 cells, either treated or not with 1% Triton X-100. *SDS*-PAGE was performed on a 15% polyacrylamide gel and analysis of Cx43 and the fragments resultant from Trypsin digestion was further performed by WB. **(b)** Immunoprecipitation (IP) of Cx43, in exosomal extracts from HEK-293V5Cx43 cells, either lysed (150 mM NaCl, 50 mM Tris-HCl, 10 mM DTT, 0.2% Saponin and 1% SDS) or intact. The same amount of total protein (5 μg) was used for inputs and for IP. **(c)** IP of Cx43-WT or Cx43-E2HA, in exosomal extracts from HEK-293A cells, previously transfected with the indicated constructs. IP was performed in lysed or intact exosomes. Inputs represent 10% of total protein used for IP.

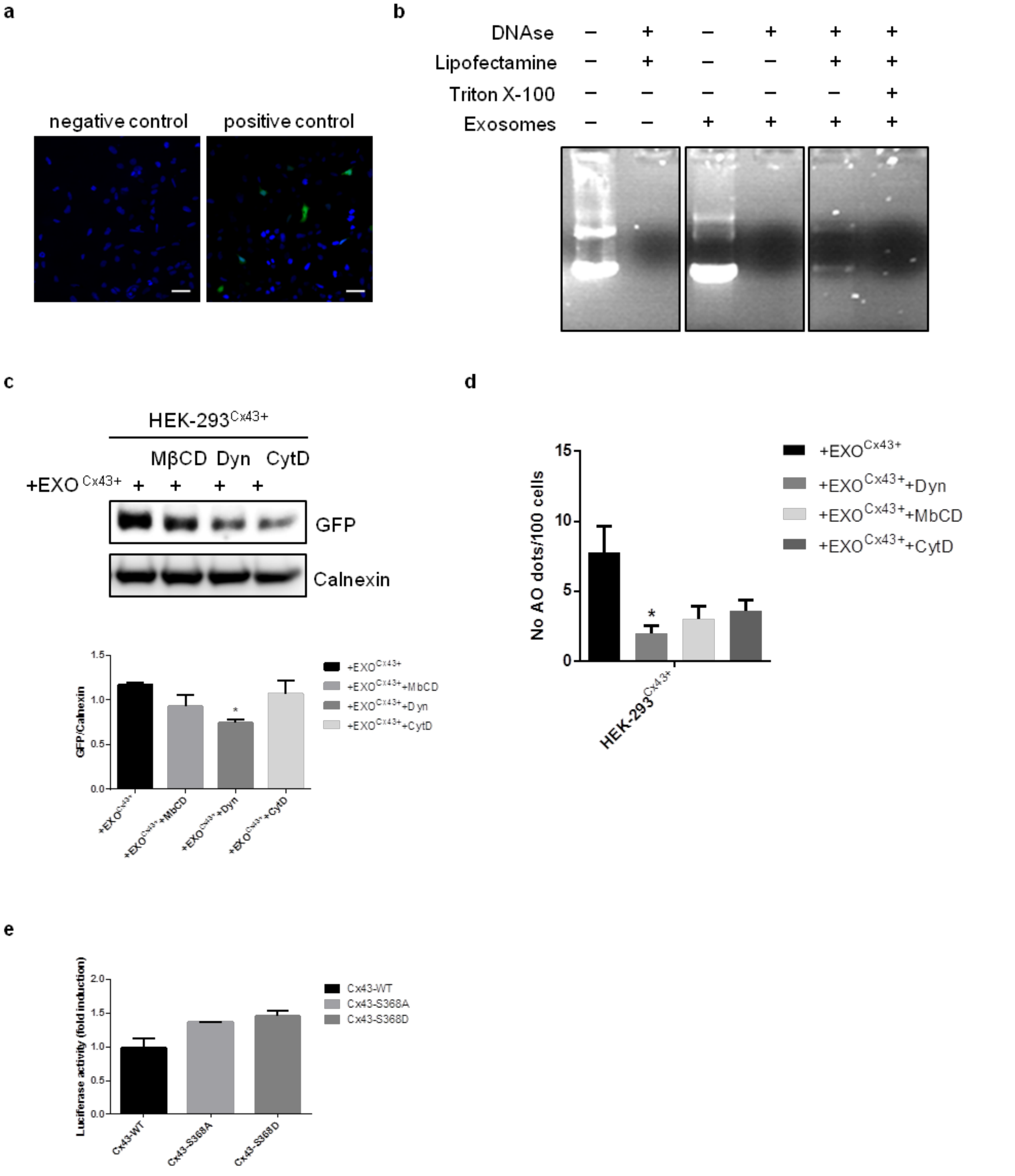

Supplementary Figure 4 – Exosomal Cx43 facilitates DNA/RNA transfer into target cells trough endocytosis. According to the manufacturer, Lipofectamine 2000 is a cationic lipid transfection reagent that forms a complex with negatively charged nucleic acids. Therefore, DNA is not encapsulated within liposomes, but rather a complex is formed between negatively charged DNA and positively charged liposomes. Thus, to attest the efficiency of the purification procedure in experiments involving chemical

loading of exosomes with heterologous DNA, Lipofectamine-DNA miceles were incubated with DNAse I and further ultra-filtrated through a 100-kDa filter. DNA that is not incorporated into exosomes is expected to be degraded by the DNAse. **(a)** Filtrates were co-cultured with recipient cells (negative control). Positive control was generated by standard transfection procedures, using Lipofectamine. **(b)** Lipofectamine-DNA miceles were incubated with DNAse I and further ultra-filtrated through a 100-kDa filter. Filtrates were subjected to agarose gel electrophoresis of DNA to verify DNA integrity. **(c)** 2.5 µg Exo^{Cx43+} were loaded with DNA encoding for GFP, after which were added to recipient cells, HEK-293^{Cx43+}. Cells were treated or not with endocytic inhibitors for 30 min. Representative WB shows the levels of GFP expression (n=3), *p<0.05. **(d)** 5 µg Exo^{Cx43+} were labeled with Acridine Orange (AO), after which 1.25 µg were added to recipient cells, HEK-293^{Cx43+}, treated with chemical inhibitors of endocytosis, where indicated. Graph shows the fluorescence microscopy analysis using Image J. Values represent the count of AO dots/100cells (n=3), *p<0.05.

SUPPLEMENTARY EXPERIMENTAL PROCEDURES

Antibodies and chemicals

Goat polyclonal antibodies against Cx43 (C-terminal epitope, AB0016), CD63 (AB0047), Calnexin (AB0041) and GFP (AB0020) were obtained from Sicgen. Rabbit polyclonal antibodies against C-terminal of Cx43 (H-150), Flotillin-1 (H-104), Alix (1A12) and mouse monoclonal antibodies against CD63 (MX-49.129.5), HA-probe (F-7, SC-7392) were obtained from Santa Cruz Biotechnology. Mouse monoclonal antibodies against Tsg101 (4A10) were obtained from Abcam. Mouse monoclonal antibodies against V5 were obtained from Life Technologies. Rat anti-Hsc70 (1B5) was obtained from Enzo life sciences. Secondary antibodies goat anti-rabbit IgG-gold 15 nm and goat anti-mouse IgG-gold 5 nm were obtained from BBInternational Solutions. 4’,6-Diamidino-2-phenylindole dihydrochloride (DAPI) and PKH26 Fluorescent Cell Linker was obtained from Sigma-Aldrich.

Cell culture

The myoblast cell line H9c2 (Sigma-Aldrich) and the HEK (human embryonic kidney)-293A cell lines were cultured in DMEM (Life Technologies) supplemented with 10% fetal bovine serum (FBS), Penicillin/Streptomycin (100 U/ml:100 µg/ml) and 1% GlutaMax, as well as transformed HEK-293V5Cx43 and HEK-293GFPCx43 cells, stably overexpressing V5-Cx43 and GFP-Cx43[1]. To create the HEK-293A cell line stably expressing V5 or GFP-Cx43, cells were transduced with the lentiviral vector pLenti6-prom-CMV-(V5 or GFP)-C1-Cx43. After 1 day, 8 μg/ml blasticidin was added to select the transduced cells. The retinal pigment epithelium cell line ARPE-19 (LGC Promochem) was cultured in Ham's F12/DMEM (1:1) supplemented with 10% FBS, Penicillin/Streptomycin (100 U/ml:100 µg/ml) and 1% GlutaMax. The cardiomyocyte cell line HL-1, kindly provided by Dr William C. Claycomb[2] was cultured in Claycomb medium (Sigma-Aldrich) supplemented with 10% FBS, Penicillin/Streptomycin (100 U/ml:100 µg/ml), 0.1 mM Norepinephrin and 2 mM L-Glutamine. All cell lines were cultured at 37°C under 5% CO_2.

Plasmid constructs

Plasmids expressing V5-Cx43 were generated by cloning Cx43 cDNA into a pENTR vector containing the V5 tag[1]. Site-directed mutagenesis was performed to generate the V5-Cx43-S368A and S368D mutants using the pENTR-V5-Cx43 plasmid mentioned above. Correct mutagenesis was verified by sequencing the constructs. pcDNA3.2/DEST/hCx43-E2HA-stop was a gift from Robin Shaw (Addgene #40909)[3].

Animal models

Wistar rats were handled according to the EU guidelines for the use of experimental animals (86/609/EEC). Experiments were approved by the Ethic Committee of the Faculty of Medicine, University of Coimbra, Portugal. For Langendorff experiments, 12-week-old rats (400 ± 25 g) were anaesthetised using 85 mg/kg ketamine and 10 mg/kg xylazine and heparinized. Hearts were isolated and perfused for 30 min in Langendorff apparatus (perfusion pressure of 70 mmHg;constant flow rate of 15 ml/min), with modified Krebs-Henseleit (KH) buffer (in mM: 118 NaCl, 25 NaHCO3, 4.7 KCl, 1.2 MgSO4, 1.2 KH2P04, 10 HEPES, 1.25 CaCl2 and 10 glucose, pH 7.49), equilibrated with 95% O_2/5%

CO_2 at 37ºC, as previously described[4]. Exosomes were isolated from the perfusate by differential ultracentrifugation. For organotypic cultures, hearts from post-natal day 7 rats were excised and ventricles sagitally sliced (1 mm thickness) by the use of a rodent heart matrix (Harvard Apparatus). Heart slices were transferred to a semiporous membrane (Millicel-CM 0.4 mm membrane, Millipore), and inserts placed in 6-well plates with 1 mL DMEM with 2 mM GlutaMAX, 10% FBS and Penicillin/Streptomycin (100 U/mL:100 µg/mL), in a humidified atmosphere containing 5% CO_2, at 37°C[5]. Cultures were maintained in exosome-depleted medium for 48h, after which exosomes were isolated by differential ultracentrifugation.

Exosome purification

Exosomes derived from cultured cells were isolated from conditioned medium. Cells were cultured in exosome-depleted medium, prepared accordingly to Lässer and collegues[6]. Briefly, 50% FBS was ultracentrifuged at 120,000*g* for 16h, after which supernatants were diluted to a final concentration of 10% FBS in the appropriate culture medium. After incubation for 24 or 48 h, as indicated, medium was collected and exosomes were isolated by ultracentrifugation, as previously described[6]. Briefly, the harvested supernatant was subjected to differential centrifugation at 4°C, starting with a centrifugation at 300*g,* for 10 min, followed by 20 min at 16,500*g*. To thoroughly remove cellular debris and larger particles, the supernatant was filtered with a 0.22-μm filter unit, after which it was ultracentrifuged at 120,000*g*, for 70 min. The resultant pellet was washed with PBS, and after ultracentrifugation, exosomes were resuspended in PBS. On average, for the cell lines used in this study, 10 µg of purified exosomes were obtained from 20 million cells. The same method was applied for exosomal purification from organotypic heart slices and heart perfusates. Human blood samples were withdrawn from healthy volunteers and exosome isolation was performed as previously described [7]. 3 ml plasma were diluted with an equal volume of PBS and centrifuged 30 min at 2,000*g*, at 4°C. Supernatant was ultracentrifuged for 45 min at 12,000*g*, and the resultant supernatant was further ultracentrifuged for 2 hr, at 110,000*g*. Pellets were resuspended in 1 ml PBS, transferred to a new tube, diluted in PBS and filtered through a 0.22-µm filter. Subsequently, two ultracentrifugations of 110,000*g* for 70 min were performed.

Western Blot (WB)

After exosome isolation, protein concentration was determined using Pierce™ BCA Protein Assay Kit, after which exosomal extracts were denatured with SDS sample buffer containing either reducing (Laemmli buffer) or non-reducing agents, where indicated [7]. Samples were then heated at 95°C, for 5 min, separated by SDS-PAGE, transferred to nitrocellulose membranes, and probed with appropriate antibodies. Unless stated otherwise, Cx43 antibodies from Sicgen were used in all WB analysis. The same experimental approaches was used in cell lysates, when applicable[1].

Targeted SWATH for detection of Cx43 in exosomes

A specific library of precursor masses and fragment ions for Cx43 was created. Briefly, a sample enriched in Cx43 (an immunopurification sample of Cx43 with a V5 tag – HEK-293V5Cx43) was used in Information Dependent Acquisition (IDA) experiments. The immunopurified sample was processed by *in gel* digestion. Gel region from 36 to 60 kDa was sliced in small pieces and destained with 50 mM ammonium bicarbonate and 30% acetonitrile. In-gel digestion was performed, with 10 ng/µL trypsin in 10 mM ammonium bicarbonate. Peptides were extracted with 30%, 50%, and 98% acetonitrile in 1% formic acid, dried by rotary evaporation under vacuum, and resuspended in 2% acetonitrile and 0.1% formic acid. Peptides were resolved by liquid chromatography (nanoLC Ultra 2D, Eksigent) on a ChromXPTM C18AR reverse phase column (300 µm ID × 15cm length, 3 µm particles, 120 Å pore size, Eksigent) at 5µL/min. Peptides were eluted into the mass spectrometer with an acetonitrile gradient in 0.1% FA (2% to 35% ACN, in a linear gradient for 25 min), using an electrospray ionization source (DuoSpray TM Source, ABSciex). The mass spectrometer (Triple TOFTM 5600 System; ABSciex) was programmed for information dependent acquisition (IDA) scanning full spectra (350-1250 m/z), followed by 20 MS/MS on multiple charged ions (+2 to +5) and performed one MS/MS before adding those ions to the exclusion list for 15 s (mass spectrometer operated by Analyst® TF 1.6, ABSciex). Peptide identification was performed using three different search engines: MASCOT (Matrix Science), PEAKS studio (v4.5, SP2, Bioinformatics Solutions Inc.) and Protein Pilot software (v4.5, ABSciex). Positive identifications were considered when proteins had more than one peptide hit with individual score above

95% of confidence level, or based on a single peptide hit with a minimum individual score of 95% and a minimum sequence tag of 3 amino acids (4 consecutive peaks in the MS/MS spectrum). The library for Cx43 was built with the 8 peptides identified between the three search engines plus 4 iRTs peptides (used as internal standards), for the peptides identified in MASCOT or PEAKS the fragment ions were manually picked.

A sample enriched in Cx43 (positive control) and the sample of exosomes, both spiked with iRT peptides, were digested as described above and analyzed on an AB Sciex 5600 TripleTOF using the optimized SWATH (Sequential Windowed data independent Acquisition of the Total High-resolution Mass Spectra) acquisition method [8]. Peptide separation was performed as described above. For SWATH-MS–based experiments, the mass spectrometer was operated in a looped product ion mode. The instrument was specifically tuned to allow a quadrupole resolution of 10 m/z mass selection. A set of 12 windows was constructed covering the precursor mass range of the peptides identified for Cx43 and for the iRT peptides (Supplementary Table 2). A 250 ms survey scan (350-1500 m/z) was acquired at the beginning of each cycle for instrument calibration and SWATH MS/MS spectra were collected from 100–1500 m/z for 250 ms resulting in a cycle time of 3.3 s. The collision energy for each window was determined according to the calculation for a charge 2+ ion centered upon the window with a collision energy spread of 15. Data processing was performed using SWATH™ processing plug-in for PeakView (v2.0.01, ABSciex). Briefly, for the targeted peptides, the fragment ions, up to 5, were automatically selected following the criteria described in Lambert et al. [9]. In SWATH™ Acquisition data, peptides are confirmed by finding and scoring peak groups, which are a set of fragment ions for the peptide. Peak groups were scored following the criteria described previously [9] and peptides that met the 1% FDR threshold were considered.

Immunoprecipitation (IP)

IP of exosomal Cx43 was performed in 2.5 µg exosomes lysed in 150 mM NaCl, 50 mM Tris-HCl, 10 mM DTT, 0.2% saponin and 1% SDS. Exosomal extracts were sonicated (4 pulses of 2’’, 180 watts), denatured at 95ºC for 5 min, and then diluted to a final concentration of 0.2% SDS. In parallel, intact

exosomes were used for IP of Cx43. Briefly, protein G was incubated with goat antibody directed against Cx43 (Sicgen) or with mouse antibody against HA (Santa Cruz), for 1 h at 4°C. Nonspecific antibodies were used as controls. Incubation with exosomal extracts was further performed, for 3h at 4°C. Protein G-pellets were washed 3 times in lysis buffer, resuspended in Laemmli buffer and denatured at 95°C, for 5 min, followed by WB analysis.

Exosomal RNA transfer into target cells

HEK-293^{Cx43+} cells were cultured in exosome-depleted medium for 48h, after which exosomes were obtained by ultracentrifugation from cell culture supernatants. For exosomal RNA transfer assays, 5μg of Exo^{Cx43+} exosomes were labeled with Acridine Orange (AO) (Calbiochem) to a final concentration of 20 μM. AO is a cell-permeable, cationic fluorescent dye that interacts with DNA and RNA by intercalation or electrostatic attractions. Staining solution was incubated with exosomes for 90 min at RT, protected from light. Excess dye was washed using Exosome Spin Columns. 1.25 μg of exosomes were co-cultured for 30 min at 37ºC with HEK-293^{Cx43+} recipient cells grown on 96 well glass bottom plates, treated or not with chemical inhibitors of endocytosis [10 mM Methyl-β-cyclodextrin (Sigma-Aldrich), 80 μM Dynasore (Sigma-Aldrich), 25 μM Cytochalasin D (Sigma-Aldrich)]. Cells were fixed with 4% PFA, rinsed in PBS and images were collected by fluorescence microscopy using a Zeiss Axio Observer.Z1 (Carl Zeiss). Quantification of internalized exosomal RNA (AO dots) was performed using Image J software.

SUPPLEMENTARY REFERENCES

1. Catarino, S., Ramalho, J. S., Marques, C., Pereira, P. & Girão, H. Ubiquitin-mediated internalization of connexin43 is independent of the canonical endocytic tyrosine-sorting signal. *Biochem. J.* **437,** 255–67 (2011).

2. Claycomb, W. C. *et al.* HL-1 cells: A cardiac muscle cell line that contracts and retains phenotypic characteristics of the adult cardiomyocyte. *Proc. Natl. Acad. Sci.* **95,** 2979–2984 (1998).

3. Smyth, J. W. *et al.* Actin cytoskeleton rest stops regulate anterograde traffic of connexin 43 vesicles to the plasma membrane. *Circ. Res.* **110,** 978–89 (2012).

4. Martins-Marques, T. *et al.* Ischemia-induced autophagy leads to degradation of gap junction protein Connexin43 in cardiomyocytes. *Biochem. J.* (2015). doi:10.1042/BJ20141370

5. Habeler, W. *et al.* An in vitro beating heart model for long-term assessment of experimental therapeutics. *Cardiovasc. Res.* **81,** 253–9 (2009).

6. Lässer, C., Eldh, M. & Lötvall, J. Isolation and characterization of RNA-containing exosomes. *J. Vis. Exp.* **59,** 1–6 (2012).

7. Théry, C., Clayton, A., Amigorena, S. & Raposo, G. Isolation and Characterization of Exosomes from Cell Culture Supernatants. *Curr. Protoc. Cell Biol.* **3,** 1–29 (2006).

8. Gillet, L. C. *et al.* Targeted data extraction of the MS/MS spectra generated by data-independent acquisition: a new concept for consistent and accurate proteome analysis. *Mol. Cell. Proteomics* **11,** 1–17 (2012).

9. Lambert, J.-P. *et al.* Mapping differential interactomes by affinity purification coupled with data-independent mass spectrometry acquisition. *Nat. Methods* **10,** 1239–45 (2013).
